# Endogenous testosterone density is an independent predictor of pelvic lymph node invasion in high-risk prostate cancer: results in 201 consecutive patients treated with radical prostatectomy and extended pelvic lymph node dissection

**DOI:** 10.1007/s11255-022-03103-w

**Published:** 2022-01-19

**Authors:** Antonio Benito Porcaro, Alessandro Tafuri, Andrea Panunzio, Giovanni Mazzucato, Clara Cerrato, Sebastian Gallina, Alberto Bianchi, Riccardo Rizzetto, Nelia Amigoni, Emanuele Serafin, Francesco Cianflone, Rossella Orlando, Ilaria Gentile, Filippo Migliorini, Stefano Zecchini Antoniolli, Giacomo Di Filippo, Matteo Brunelli, Vincenzo Pagliarulo, Maria Angela Cerruto, Alessandro Antonelli

**Affiliations:** 1 Department of Urology, University of Verona, Azienda Ospedaliera Universitaria Integrata, Verona, Italy; 2 Department of Urology, Vito Fazzi Hospital, Lecce, Italy; 3 Department of Neuroscience, Imaging and Clinical Sciences, G. D’Annunzio University, Chieti, Italy; 4 Department of General and Hepatobiliary Surgery, Azienda Ospedaliera Universitaria Integrata, University of Verona, Piazzale Stefani 1–37126, Verona, Italy; 5 Department of Pathology, University of Verona, Azienda Ospedaliera Universitaria Integrata, Verona, Italy

**Keywords:** Prostate cancer, High risk prostate cancer, Radical prostatectomy, Extended pelvic lymph node dissection, Pelvic lymph node invasion, Endogenous testosterone, Prostate volume, Endogenous testosterone density, Prostate specific antigen, Prostate specific antigen density, Percentage of biopsy positive cores density, Tumor load density

## Abstract

**Objective:**

To evaluate the influence of endogenous testosterone density (ETD) on pelvic lymph node invasion (PLNI) in high risk (HR) prostate cancer (PCa) treated with radical prostatectomy (RP) and staged with extended pelvic lymph node dissection (ePLND).

**Materials and methods:**

ETD was evaluated as the ratio of endogenous testosterone (ET) on prostate volume (PV). HR-PCa was assessed according to the European Association of Urology (EAU) system. The association of ETD and other routinely clinical factors (BPC: percentage of biopsy positive cores; PSA: prostate specific antigen; ISUP: tumor grade system according to the International Society of Urologic Pathology; cT: tumor clinical stage) with the risk of PLNI was assessed by the logistic regression model.

**Results:**

Overall, 201 out of 805 patients (24.9%) were classified HR and PLNI occurred in 42 subjects (20.9%). On multivariate analysis, PLNI was independently predicted by BPC (OR 1.020; 95% CI 1.006–1.035; *p* = 0.019), ISUP > 3 (OR 2.621; 95% CI 1.170–5.869; *p* = 0.019) and ETD (OR 0.932; 95% CI 0.870–0.999; *p* = 0.045). After categorizing continuous clinical predictors, the risk of PLNI was independently increased by ETD up to the median (OR 2.379; 95% CI 1.134–4.991; *p* = 0.022), BPC > 50% (OR 3.125; 95% CI 1.520–6.425; *p* = 0.002) as well as by ISUP > 3 (OR 2.219; 95% CI 1.031–4.776; *p* = 0.042).

**Conclusions:**

As ETD measurements decreased, patients were more likely to have PLNI. In HR disease with PLNI, the influence of PCa on ETD should be addressed by higher level studies.

**Supplementary Information:**

The online version contains supplementary material available at 10.1007/s11255-022-03103-w.

## Introduction

Prostate cancer (PCa) is the second most diagnosed tumor in the aging male and its management has become a pivotal health problem in developing countries [1, 2]. According to European Association of Urology (EAU) and National Comprehensive Cancer Network (NCCN) guidelines, three PCa categories can be identified as related to the risk of developing metastases [1, 2]. Among these, the high risk (HR) class is the most controversial for both classification among international societies and treatment which may include, for localized disease, radical prostatectomy (RP) with extended pelvic lymph node dissection (ePLND), radiotherapy (RT) and androgen suppression therapy and or a combination of multimodality treatments [1, 2]. When surgery is opted, an accurate anatomical staging using extended template is a critical issue for the high risk of pelvic lymph node invasion (PLNI) [1, 2]. However, residual disease may occur in the prostatic fossa and/or loco regional and retroperitoneal lymph nodes explaining prostate specific antigen (PSA) persistence or recurrence after surgery is a non-complete LND has been performed [1–3].

PCa has been related to several risk factors including genetic, dietary, environmental, physical (obesity, metabolic syndrome) and hormonal (hypogonadism) factors, as well [1, 2]. Endogenous testosterone (ET) is an important factor for evaluating prostate growing disorders including either benign prostatic hyperplasia (BPH) and PCa, which may also coexist. Our group has greatly focused its attention of the role of ET in PCa, finding that it could be associated with several unfavorable prognostic factors [4–6]. In low risk PCa, we have recently shown that ET density (ETD), defined as the ratio of ET on prostate volume (PV), was an independent predictor of the risk of high tumor load (TL), which associated with unfavorable disease in the surgical specimen [7]. In the present study, we wanted to address the hypothesis that ETD may relate to PLNI in HR disease treated with RP and ePLND.

## Materials and methods

### Study population

The study was approved by Institutional Review Board. Informed consent was obtained by all subjects. Data were collected prospectively but retrospectively evaluated. In a period ranging from November 2014 to December 2019, 805 consecutive PCa patients who were not under androgen blockade had ET (nmol/L) measured at our lab before surgery and the test was performed at least 1 month after biopsies between 8.00 and 8.30 a.m. by radioimmunoassay. PSA (ng/mL), age (years), body mass index (BMI; kg/m^2^), PV (mL) and percentage of BPC, the ratio of positive and total taken cores (%), were evaluated in each case. PV was calculated by TRUS standard methods. Biopsies performed elsewhere were assessed for number of cores taken, tumor grade and PV. In our Institution, the 14-core trans perineal technique was used [8]. In each case, the ratios of BPC, PSA and ET with PV were calculated, and relative densities indicated as BPCD (%/mL), PSAD (ng/ml^2) and ETD (nmol/ (dL x mL)), respectively. Clinical staging was assessed by the 2017 version of the TNM system (8th edition) with clinical T stage only referring to DRE findings and patients were classified into risk classes, as recommended by EAU guidelines [1]. Surgery, which was delivered by robot-assisted (RARP) or open approach (ORP), was performed by experienced surgeons. Extended PLND was performed according to guidelines [1, 2]. Nodal packets were submitted in separate packages according to a standard anatomical template including external iliac, internal iliac plus obturator, Marcille’s common iliac, and Cloquet’s nodal stations, bilaterally [9, 10]. Specimens including prostate and dissected lymph nodes were placed into formalin and evaluated by the dedicated pathologist. Prostates were weighted and tumors were graded according to the ISUP system [1, 2]. Tumor quantitation was assessed as TL, which was defined as percentage of prostate involved by cancer; specifically, our dedicated pathologist assessed tumor quantitation by visual estimation of all the glass slides after all microscopically identifiable foci of carcinoma have been circled with a marked pen, as considered by ISUP association [11]. TL density (TLD) was calculated as the ratio of TL on prostate weight (%/gr). Surgical margins were stated positive when cancer invaded the inked surface of the specimen. Removed lymph nodes were assessed for number and cancer invasion. Surgical specimens were staged by the 2017 version of the TNM system (8th edition), accordingly [1, 2].

### Statistical methods

Continuous variables were measured for means (standard deviation, SD) and medians (interquartile range, IQR). Categorical factors were assessed for frequencies (percentages). Associations of ETD with clinical and pathological factors including PLNI were tested by the linear regression model (univariate and multivariate analysis). Appropriate biplots were computed, accordingly. Associations of ETD and other clinical/pathological factors with the risk of PLNI were assessed by the logistic regression model (univariate and multivariate analysis); furthermore, the discriminant power of significant continuous predictors were assessed by receiver operating characteristic (ROC) curve analysis with relative area under the curve (AUC). The software used to run the analysis was IBM-SPSS version 26. All tests were two-sided with *p* < 0.05 considered to indicate statistical significance.

## Results

### Demographics of the patient population

Overall, 201 out of 805 PCa patients (24.9%) were classified as HR class by EAU system [1]. Accordingly, ISUP grade groups 1 to 3 were diagnosed in 87 cases (43.3%) and grade 4 to 5 in 114 subjects (56.7%). Tumors were clinically staged as cT1c in 90 cases (44.8%) and cT2/3 in 111 (55.2%); furthermore, suspected nodal involvement was assessed in 34 cases (16.9%). Mean (SD) and median (IQR) PSA were 12.5 (12.6) ng/dL and 7.5 (5.4–14.1) ng/mL, respectively. The median (IQR) percentage of BPC was 50 (26.5–70). According to the preoperative physical status system by American Society of Anesthesiologists (ASA), 19 patients were ASA I (9.5%), 161 ASA II (80.1%) and 21 ASA III (10.4%), as well. Surgery was delivered by the robotic approach in 171 (85.1%) patients. Further details are reported in Table 1. In the surgical specimen, ISUP grade group < 4 was detected in 86 subjects (42.8%), while ISUP > 3 was present in 115 cases (57.2%); as such, tumor upgrading occurred 27 out of 87 ISUP < 4 patients (31%), while tumor downgrading was assessed in 26 out of 114 ISUP > 3 cases (22.8%). Cancers extended beyond the prostate in 75 cases (37.3%) with extracapsular extension in 51 subjects (25.4%) and seminal vesicle invasion in 24 patients (11.9%). Positive surgical margins were detected in 75 subjects (37.3%). The median (IQR) of counted lymph was 26 (20–32). Overall, PLNI occurred in 42 patients (20.9%).

**Table 1:** Demographics of 201 high risk prostate cancer patients treated with radical prostatectomy and extended pelvic lymph node dissection

Continuous variables	Mean (SD)	Median (IQR)
Age (years)	65.9 (6.1)	67 (61.7–71)
Body mass index; BMI (kg/m^2)	26.2 (3.3)	25.6 (24–28.7)
Endogenous testosterone; ET (ng/dL)	450.4 (132.4)	445 (364.1–537.2)
ET density; ETD (ng/(dL x mL))	11.9 (7.38)	10 (7.1–15.1)
Prostate specific antigen; PSA (ng/mL)	12.5 (15.6)	7.5 (5.4–14.1)
PSA density; PSAD (ng/(mL x mL))	0.29 (0.33)	0.19 (0.12–0.34)
Prostate volume; PV (mL)	44.7 (18.4)	41 (30–57)
Percentage of biopsy positive cores; BPC (%)	48.9 (26.8)	50 (26.5–70)
BPC density; BPCD (%/mL)	1.2 (0.9)	1.1 (0.5–1.7)
Prostate weight; PW (grams; gr)	57.6 (20.2)	54.5 (44.3–70)
Tumor load; TL (%)	29.8 (21.8)	25 (15–40)
Tumor load density; TLD (%/gr)	0.5 (0.4)	0.43 (0.21–0.70)
Number od dissected lymph nodes; LN (n)	26 (9.4)	26 (20–32)
Categorical variables	Number (%)	
ISUP at biopsy		
ISUP 1	25 (12.4)	
ISUP 2	36 (17.9)	
ISUP 3	26 (12.9)	
ISUP 4	93 (46.3)	
ISUP 5	21 (10.5)	
Clinical T stage (cT)		
cT1c	90 (44.8)	
cT > 1	111 (55.2)	
Clinical nodal stage (cN)		
cN0	167 (83.1)	
cN1	34 (16.9)	
ISUP at pathology		
ISUP = 1	8 (4)	
ISUP = 2	28 (13.9)	
ISUP = 3	50 (24.9)	
ISUP = 4	66 (32.8)	
ISUP = 5	49 (24.4)	
Pathologic tumor stage (pT)		
pT2	120 (62.7)	
pT3a	51 (25.4)	
pT3b	24 (11.9)	
Surgical margins status (SM)		
Negative (NSM)	126 (62.7)	
Positive (PSM)	75 (37.3)	
Pathologic nodal stage (pN)		
pN0	159 (79.1)	
pN1	42 (20.9)	

### Associations of ETD with clinical and pathological features

Associations of ETD with clinical and pathological parameters were investigated by linear regression methods and reported in supplementary Table S1. On univariate analysis, ETD related positively to ET, PSAD, BPCD, TLD but inversely to PV, PW and PLNI. The positive association between ETD and ET is shown in supplementary Figure S1: as ET levels increased, ETD values also increased, accordingly. No significant associations were found out with the other remaining factors. On multivariate analysis model I (without TLD), ETD associated positively with BPCD (regression coefficient, *b* = 3.260; 95% CI 2.325; 4.195; *p* < 0.0001) and inversely with PLNI (*b* = − 3.324; 95% CI − 5.557; − 1.092; *p* = 0.004). On multivariate model II (excluding BPCD), ETD positively associated with PSAD (*b* = 3.790; 95% CI 0.795; 6.785; *p* = 0.013) and TLD (*b* = 2.995; 95% CI 0.859; 5.130; *p* = 0.006) but inversely with PLNI (*b* = − 4.023; 95% CI − 6.540; − 1.507; *p* = 0.002). Supplementary Figure S2 illustrates the positive association of ETD with PSAD: as PSAD increased ETD incremented accordingly but lower ETD values were detected in tumors associating with PLNI. Supplementary Figure S3 shows the positive association of ETD with TLD: as TLD incremented ETD increased accordingly; however, tumors associating with PLNI showed lower mean levels of ETD when compared with cancers without, as well.

Associations of ETD with TLD among EAU risk categories were also reported in supplementary Table S3 and illustrated in supplementary Figure S4. In the overall patient population including 805 patients, ETD and TLD were highly correlated to each other (Pearson’s correlation coefficient, *r* = 0.214; *p* < 0.0001). As EAU risk categories increased, TLD increased, accordingly. ETD did not show any significant distribution among EAU risk classes; however, it was an independent predictor of TLD together with EAU risk classes, as well. As ETD increased, TLD increased but increments for higher for intermediate and high-risk classes when compared to the low-risk class. Furthermore, as shown by the model, for same values of TLD (as an example TLD = 1), measurements of ETD were significantly lower for the high-risk class meaning lower ET levels thus addressing features of aggressive disease.

### Associations of ETD with of PLNI

**Fig. 1:**
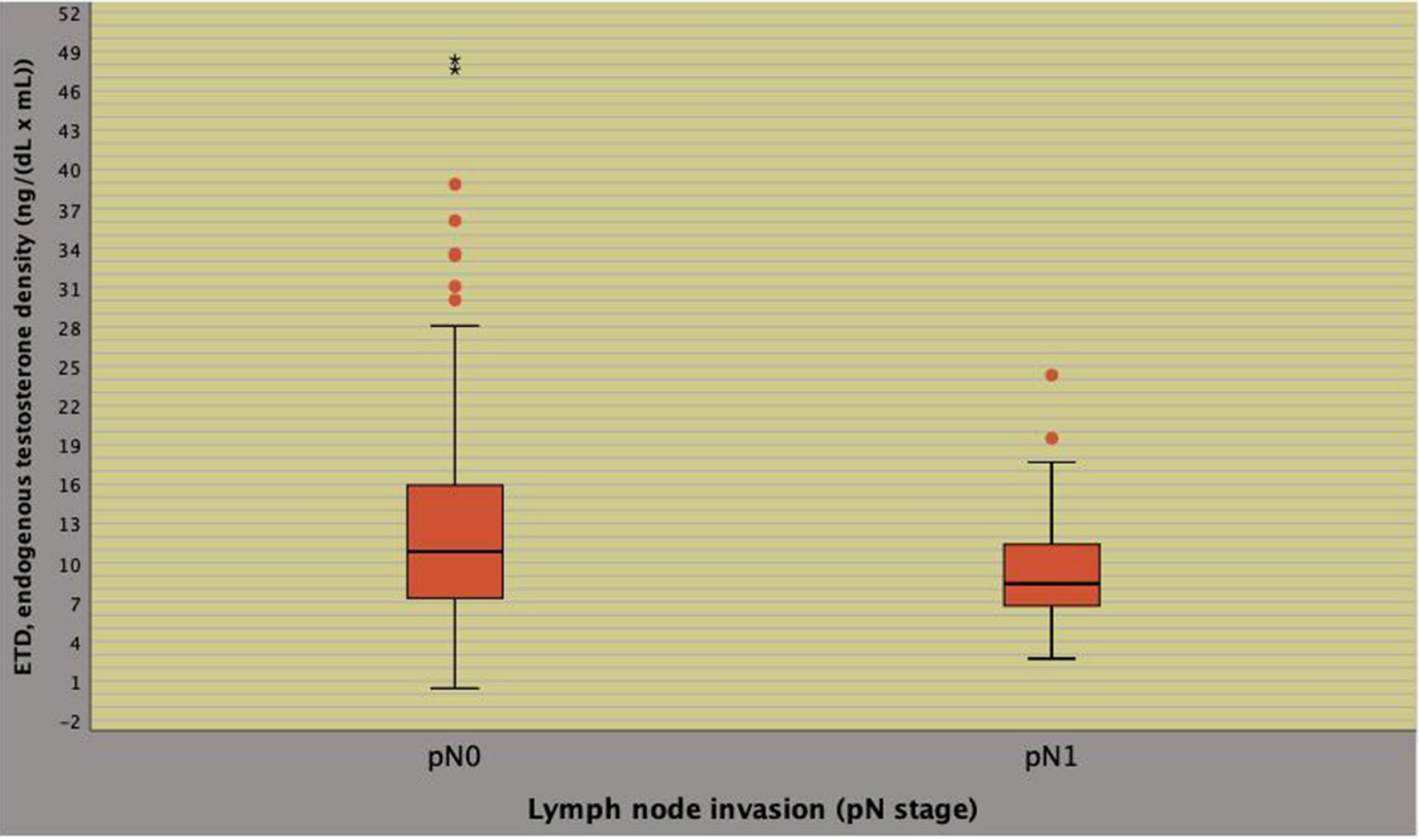
Inverse association between endogenous testosterone density (ETD) and risk of pelvic lymph node invasion (PLNI). Significantly lower median levels of ETD were detected in tumors associated with PLNI compared with cancers without (8.4 vs 10.8 ng/(dL × mL); odd ratio, OR 0.931; 95% CI 0.873–0.994; *p* = 0.031). As ETD decreased, the risk of detecting PLNI increased, accordingly

**Fig. 2:**
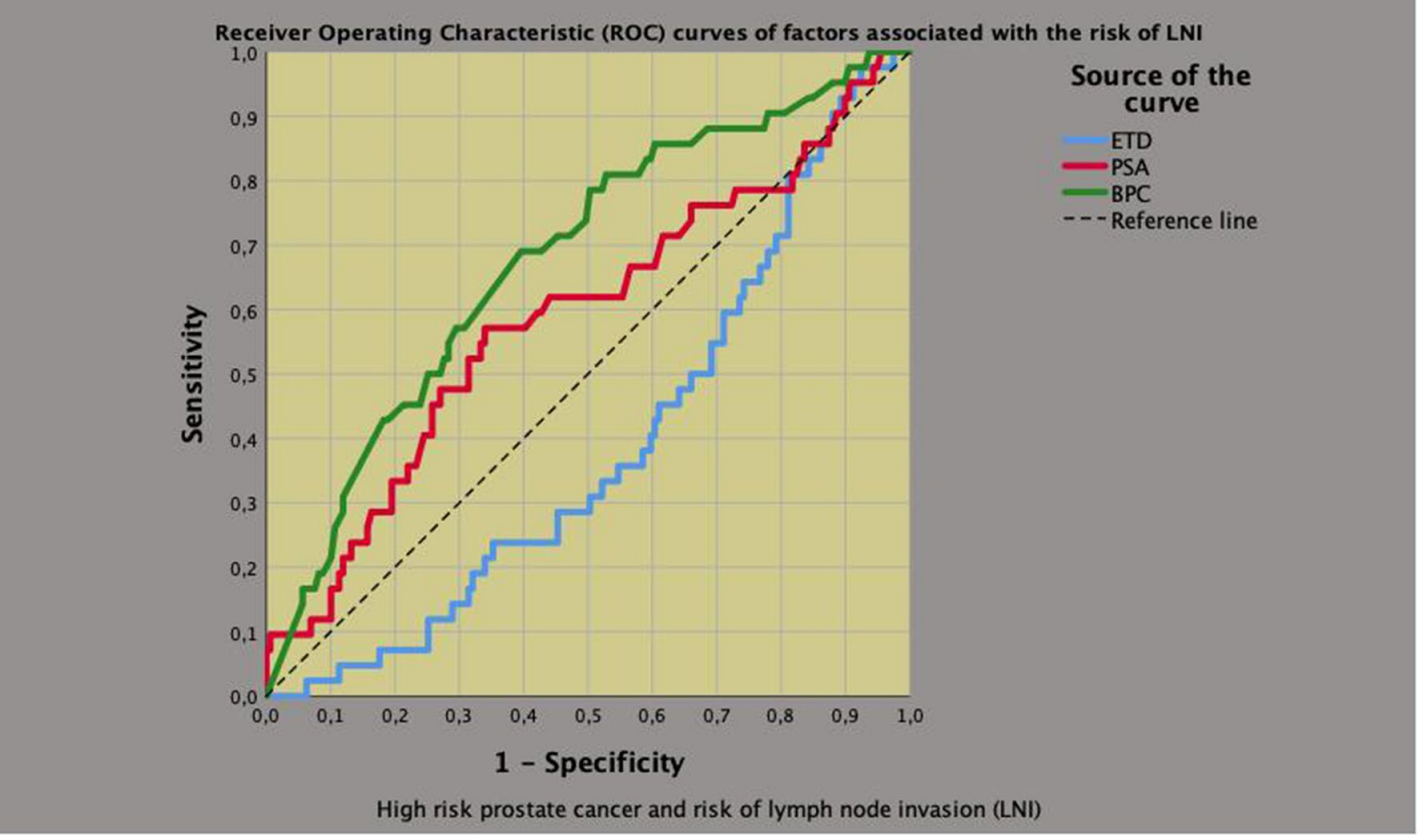
Receiver operating characteristic (ROC) curves of significant clinical continuous variables associated with the risk of pelvic lymph node invasion (PLNI), which was positive for prostate specific antigen (PSA) and percentage of biopsy positive cores (BPC), but inverse for endogenous testosterone density (ETD). The risk of PLNI increased as PSA and BPC increased as well as ETD decreased, accordingly. Area under the curves (AUC) and (95% CI) were as follows: **a** ETD: 0.389 (0.300–0.477; *p* = 0.026); **b** BPC: 0.678 (0.588–0.769; *p* < 0.0001); **c** PSA: 0.588 (0.486–0.690; *p* = 0.079)

**Fig. 3:**
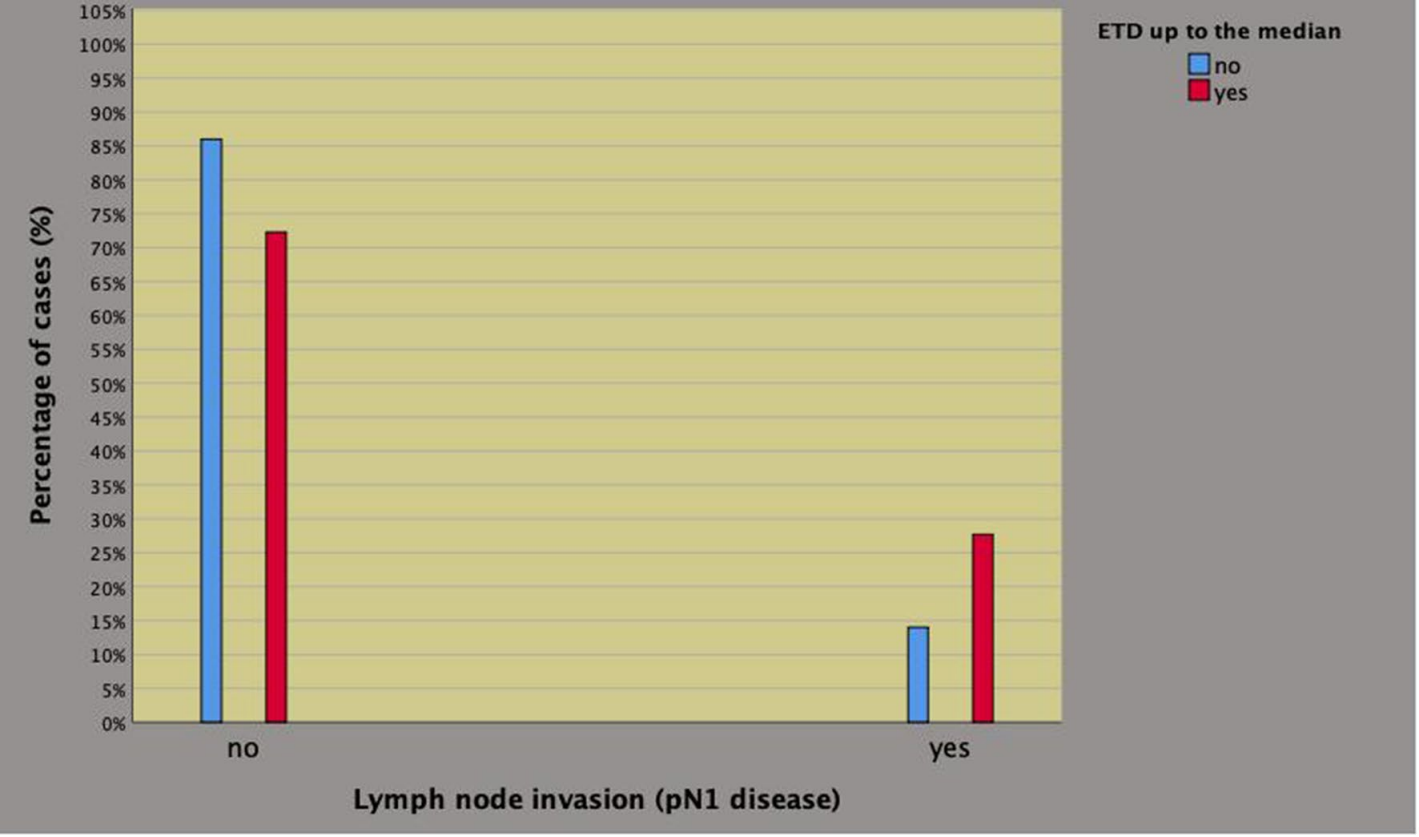
Endogenous testosterone density (ETD) associated with the risk of pelvic lymph node invasion (PLNI) in patients with high-risk prostate cancer treated with radical prostatectomy and extended pelvic lymph node dissection. Patients presenting with ETD up to the median were more likely to occult PLNI (27.7%) compared with subjects having ETD measurements above the median (14%) and the association was significant on both univariate analysis (OR 2.356; 95% CI 1.155–4.809; *p* = 0.019) as well as after adjusting for percentage of biopsy positive cores above 50% and tumor grade above 3 according to the International Society of Urologic Pathology (ISUP) system (adjusted OR 2.379; 95% CI 1.134–4.991; *p* = 0.022). See also Table 3 and results for further details

Associations of clinical and pathological factors with the risk of PLNI are reported in Table 2. On univariate analysis, the risk of PLNI was significantly predicted by PSA, BPC, ISUP > 3, cT > 1c as well as by ETD with associations being positive for the first four predictors and inverse for the latter; furthermore, PLNI was also significantly predicted by pathological factors including TLD, seminal vesicle invasion, positive surgical margins and number of dissected lymph nodes with all predictors showing positive association. Figure 1 illustrates the inverse association between ETD and risk of PLNI. As depicted, significantly lower median levels of ETD were detected in tumors associated with PLNI compared with cancers without (8.4 vs 10.8 ng/ (dL × mL); odds ratio, OR 0.931; 95% CI 0.873–0.994; *p* = 0.031); so far, as ETD decreased, the risk of detecting PLNI increased, accordingly. Figure 2 depicts ROC curves of significant clinical continuous variables associated with the risk of PLNI; as illustrated, the association was positive for PSA and BPC, but inverse for ETD. So far, the risk of PLNI increased as PSA and BPC increased as well as ETD decreased, accordingly. On multivariate analysis, PLNI was independently predicted only by BPC (OR 1.020; 95% CI 1.006–1.035; *p* = 0.019), ISUP > 3 (OR 2.621; 95% CI 1.170–5.869; *p* = 0.019) and ETD (OR 0.932; 95% CI 0.870–0.999; *p* = 0.045). Considering pathological parameters, PLNI was independently predicted by ISUP > 3, seminal vesicle invasion, positive surgical margins and number of dissected lymph nodes, as detailed in Table 2. Continuous clinical predictors of PLNI were then categorized as follows: a BPC > 50% vs < 50%; b ETD up to the median (10 ng/ (mL × mL)) versus above. Table 3 describes analysis of clinical factors associated with the risk of PLNI. On univariate analysis, all categorized factors were positive predictors of PLNI. On multivariate analysis, the risk of PLNI was independently increased by ETD up to the median (OR 2.379; 95% CI 1.134–4.991; *p* = 0.022), BPC > 50% (OR 3.125; 95% CI 1.520–6.425; *p* = 0.002) as well as by ISUP > 3 (OR 2.219; 95% CI 1.031–4.776; *p* = 0.042). As detailed in Table 3 and illustrated in Fig. 3, patients presenting with ETD up the median were more likely to occult PLNI (27.7%) compared with those with ETD above the median (14%). ETD was also tested on a multivariate model including significant pathological predictors of PLNI, as reported in supplementary Table S2. After adjusting for ISUP > 3, seminal vesicle invasion, positive surgical margins and number of counted lymph nodes, ETD up to the median was still an independent predictor of the risk of PLNI (OR 2.592; 95% CI 1.071–6.275; *p* = 0.035). Further details are described in Table S2.

**Table 2:** Factors predicting pelvic lymph node invasion (PLNI) in 201 high risk prostate cancer patients treated with radical prostatectomy and extended pelvic lymph node dissection

*Statistics*	No PLNI	PLNI	Univariate analysis		Multivariate analysis	
	Median (IQR) or frequency (%)	Median (IQR) or frequency (%)	OR (95% CI)	P value	OR (95% CI)	*P* value
Clinical model						
Age	67 (61–71)	68.5 (62.7–71)	1.036 (0.978–1.0.98)	0.232		
BMI	25.4 (23.7–28.4)	26.1 (24.2–28.7)	1.024 (0.945–1.110)	0.560		
ET	446.6 (343–548)	435.4 (361.8–493.5)	0.999 (0.997–1.001)	0.417		
ETD	**10.8 (7.2–16.3)**	**8.4 (6.7–11.7)**	**0.931 (0.873–0.994)**	**0.031**	**0.932 (0.870–0.999)**	**0.045**
PSA	**7.3 (5.3–12.3)**	**9.8 (5.8–18.7)**	**1.029 (1.006–1.053)**	**0.015**	1.023 (0.996–1.050)	0.092
PSAD	0.19 (0.11–0.34)	0.25 (0.12–0.34)	1.940 (0.784–4.802)	0.152		
PV	40 (30–56)	46.5 (38–62.4)	1.018 (1.000–1.036)	0.050		
BPC	**38 (25–60)**	**58 (38–50)**	**1.023 (1.010–1.037)**	**0.001**	**1.020 (1.006–1.035)**	**0.006**
BPCD	1.00 (0.48–1.66)	1.23 (0.62–1.71)	1.109 (0.799–1.538)	0.537		
ISUP < 4	75 (47.2)	12 (28.6)	Reference			
ISUP > 3	**84 (52.8)**	**30 (71.4)**	**2.232 (1.067–4.671)**	**0.033**	**2.621 (1.170–5.869)**	**0.019**
cT1c	77 (44.8)	13 (31)	Reference			
cT > 1c	**82 (51.6)**	**29 (69)**	**2.095 (1.015–4.322)**	**0.045**	1.713 (0.787–3.276)	0.175
cN0	130 (81.8)	37 (88.1)	Reference			
cN1	28 (18.2)	5 (11.9)	0.606 (0.219–1.675)	0.334		
No RARP	24 (15.1)	6 (14.3)	Reference			
RARP	135 (84.9)	36 (85.4)	1.067 (0.405–2.806)	0.896		
Pathological model						
PW	52 (42–67)	56 (46.5–73.2)	1.012 (0.996–1.028)	0.132		
TLD	**0.36 (0.18–0.66)**	**0.62 (0.44–1.03)**	**3.178 (1.582–6.387)**	**0.001**	1.817 (0.799–4.135)	0.155
ISUP < 4	83 (52.2)	3 (7.1)	Reference			
ISUP > 3	**76 (47.8)**	**39 (92.9)**	**14.197 (4.213–47.841)**	** < 0.0001**	**9.969 (2.779–35.768)**	** < 0.0001**
pT2	106 (66.7)	14 (33.3)	Reference			
pT3a	25 (15.7)	3 (7.1)	0.909 (0.242–3.404)	0.887		
pT3b	**28 (17.6)**	**25 (59.5)**	**6.760 (3.113–14.682)**	** < 0.0001**	**3.509 (1.564–8.188)**	**0.004**
NSM	10 (69.2)	16 (38.1)	Reference			
PSM	**49 (30.8)**	**26 (61.9)**	**3.648 (1.797–7.405)**	** < 0.0001**	**2.713 (1.147–6.421)**	**0.023**
LN (n)	**25 (19–31)**	**29 (23–34.7)**	**1.049 (1.013–1.086)**	**0.007**	**1.096 (1.017–1.180)**	**0.016**

*IQR* interquartile range; *OR* odds ratio; *CI* confidence interval; see also Table 1

Factors predicting PLNI for which a statistically significant associations was found in the univariate and multivariate analysis are reported in bold

**Table 3:** Clinical factors associated with the risk of pelvic lymph node invasion in 201 high risk prostate cancer patients

Statistics	*n* (%)	No PLNI	PLNI	Univariate analysis		Multivariate analysis	
		*n* (%)	*n* (%)	OR (95% CI)	*p* value	OR (95% CI)	*p* value
ETD							
Above the median	100 (49.8)	86 (86)	14 (14)	1		1	
Up to the median	101 (50.2)	73 (72.3)	28 (27.7)	2356 (1155–4809)	0.019	2379 (1134—4991)	0.022
BPC							
Up to 50%	128 (63.7)	110 (85.9)	18 (14.1)	1		1	
Above 50%	73 (36.3)	49 (67.1)	24 (32.9)	2993 (1490–6014)	0.002	3125 (1520—6425)	0.002
ISUP (biopsy)							
Up to 3	87 (43.3)	75 (86.2)	12 (13.8)	1		1	
Above 3	114 (56.7)	84 (73.7)	30 (26.3)	2232 (1067–4671)	0.033	2219 (1031—4776)	0.042

*PLNI* pelvic lymph node invasion; *OR* odd ratio; *CI* confidence interval; *EDT* endogenous testosterone density; *BPC* percentage of biopsy positive cores; *ISUP* International Society of Urologic Pathology tumor grade system; see also results for further details

## Discussion

Treatment of HR-PCa with RP and ePLND is actually increasing; however, uniformity in definitions and indications, which impact on oncological outcomes, are missing [12, 13]. Moreover, when comparing surgery versus radiation and androgen deprivation therapy as primary treatments of HR-PCa, evidence showing definitive superiority of either modality is lacking [13]. In clinical practice, a critical issue is assessment of PLNI. Although several validated nomograms, which refer to commonly available clinical factors, have been proposed, there is a lack of specificity for HR-PCa [1, 2]. Recently, new nomograms have been validated through mpMRI parameters including the PI-RADS score, number of PI-RADS ≥ 3 lesions, maximum diameter of index lesion and tumor stage (organ confined, extracapsular extension and seminal vesicle invasion), as well [14]. A European multicenter study while externally validating the currently available nomograms in predicting PLNI, found out that the MSKCC and Briganti 2012 nomograms were superior in predicting nodal metastases suggesting that mpMRI parameters did not add any relevant information for not being reproducible in the different centers because of the high dependency on the operator [15].So far, further simpler and newer parameters are required to validate future nomograms predicting the risk of PLNI [16]. Our study showed that ETD either a continuous or categorized variable could be an effective biomarker for assessing occult PLNI in HR-PCa according to the EAU system, independently from other clinical or pathological because of the strong association with tumor aggressive biology; however, confirmatory studies are required.

In locally advanced disease, the triangle of Marcille’s as well as Cloquet’s node are vital anatomical regions for advanced pelvic surgery in evaluating systemic nodal disease, as well [17, 18]. These findings explain why pN1 high risk patients with biochemical persistence/recurrence have their first detectable recurrence on gallium-68 prostate-specific membrane antigen positron-emission tomography (PET)/computed tomography (CT) in the prostatic fossa and/or pelvic nodes (2/3 of positive cases) as well as outside the pelvic area in distant (retroperitoneal) lymph nodes and/or bones (1/3 of cases), as well [19, 20]. So far, predictors of advanced pelvic disease are actually required in HR-PCa. Our study has specifically investigated the issue of PLNI in HR disease, which was detected in 20.9% of cases; furthermore, anatomical staging was appropriate for either extension of the template as well as for the median (IQR) number of removed nodes, which was 26 (20–32). As such, the predictivity of ETD was tested in a population who was extensively staged anatomically according to an advanced pelvic pattern including extremal territories of prostatic lymphatic system represented by Marcille’s fossa and Cloquet’s nodes, which are pivotal landmarks for evaluating PLNI in advanced disease [9, 10, 17, 18]. However, comparative studies for evaluating this issue are actually missing.

Although PCa is a hormone dependent tumor, the subject dealing with associations between ET levels and aggressive cancer features is highly controversial for controlled studies are missing and for ET not being measured on a chronic basis, as it should be [21, 22]. According to systematic reviews and meta-analysis, the association may be found positive, inverse or null [21, 22]. However, when considering associations of ET with PLNI, few studies are available; specifically, they show significant inverse associations between ET levels and risk of PLNI. Recently, we have confirmed these results showing that, in early PCa undergoing ePLND and including all risk classes according to the EAU system, there is an inverse association between ET and risk of multiple occultic metastases at loco-regional lymph nodes [23]. In the present study, we have demonstrated that ETD was an independent parameter for predicting occult metastatic disease in the high-risk population in whom as ETD decreased, the risk of PLNI increased accordingly, independently by load (BPC > 50%) and/or grade (ISUP > 3) of the tumor, as well. Although adjusting ET for prostate volumes is a novelty for HR disease, we have already investigated this feature in the low-risk category. Specifically, we have shown that ETD associated with the risk of high tumor load (percentage of cancer involving at least 20% of the gland) that predicted the risk of unfavorable disease; furthermore, although that study is not comparable with the present one for several features, either investigation showed associations of ETD with factors related to aggressive PCa, as well [7]. The results of the present study represent a novelty for the literature dealing with this topic and might have implications in clinical practice.

In HR disease with biopsy ISUP > 3, the incidence of downgrading ranges between 26 and 31%; as such, patients presenting with low ETD (up to the median) will have an increased risk of PLNI, which might be an important issue to discuss when counselling patients. Particularly, these patients should not be candidate to focal therapy [24, 25] as already suggested by international guidelines [1, 2]. Additionally, in HR locally advanced PCa treated with RP and ePLND, the risk of PLNI may be evaluated by EDT together with other routinely factors. Furthermore, pN1 patients with biochemical persistence/recurrence with low ETD measurements might occult residual disease in the pelvic and extra-pelvic nodes, independently by PET-CT PSMA results [1, 2, 19]. All these implications suggest the need of prospective controlled trials in order to assess the influence of PCa biology on either ET and ETD in the HR category, as well.

Our study has several limitations. Prostate volumes were not all measured at our institution. ET was measured only once and not on a chronic base, as recommended [22]. Central pathology review of external biopsies was not performed. Results of mpMRI were not evaluated for not being available in all patients. Genetic tests were not performed. Analysis of maximal cancer involvement of each core was not computed for not being available in all patients. Finally, the retrospective nature of the study. Our study has strengths, as well. All prostate specimens were assessed by our dedicated pathologist. ET was measured in the morning, which is the appropriate interval for evaluating the levels of the hormone, which decrease in the afternoon, as well [26]. Data were prospectively collected. It was single center study, and the patient population was homogenous for ethnicity (Caucasian) and for ET measurements, which were all performed at our laboratory.

## Conclusions

ETD, which was positively correlated to ET, inversely associated with the risk of PLNI; as ETD decreased, the risk of PLNI increased, accordingly. The influence of HR disease on either ET levels and ETD measurements needs to be evaluated by prospective multicenter studies.

## Supplementary Information

Below is the link to the electronic supplementary material.

Supplementary file1 Figure S1. Positive correlation between endogenous testosterone (ETD) and endogenous testosterone (ET): as ET levels incremented, ETD values also increased accordingly. ETD was evaluated as the ratio of ET on prostate volume. Pearson’s correlation coefficient, r = 0.619 (p < 0.0001). (JPG 53 KB)

Supplementary file2 Figure S2. Significant positive association of endogenous density (ETD) with prostate specific antigen density (PSAD): as PSAD increased, ETD incremented accordingly but lower ETD values were detected in tumors associating with pelvic lymph node invasion (PLNI), as well. ETD was evaluated as the ratio of endogenous testosterone (ET) on prostate volume (PV). (JPG 36 KB)

Supplementary file3 Figure S3. Significant positive association of endogenous testosterone density (ETD) with tumor load density (TLD): as TLD incremented, ETD increased accordingly; however, tumors associating with pelvic lymph node invasion (PLNI) showed lower mean levels of ETD when compared with cancers without, as well. ETD was evaluated as the ratio of endogenous testosterone (ET) on prostate volume, while TLD as the ratio of percentage cancer involving the prostate on prostate weight. (JPG 47 KB)

Supplementary file4 Figure S4. The boxplot of the bivariate model of ETD predicting TLD stratified by EAU risk classes (high risk versus low-intermediate classes). As ETD increased, TLD incremented, accordingly, but TLD increments were higher for EAU high risk class; moreover, as shown by the model, for same values of TLD (example TLD = 1), measurements of ETD were lower for the high-risk class, which mean lower mean levels of ET addressing features of aggressive disease. (JPG 60 KB)

Supplementary file5 (XLSX 11 KB)

Supplementary file6 (XLSX 10 KB)

Supplementary file7 (XLSX 10 KB)
