# Supplementary figures and images for: Endogenous testosterone density is an independent predictor of pelvic lymph node invasion in high-risk prostate cancer: results in 201 consecutive patients treated with radical prostatectomy and extended pelvic lymph node dissection
Source: Int Urol Nephrol. 2022 Jan 19;54(3):541–50. doi: 10.1007/s11255-022-03103-w (PMC8831287; doi:10.1007/s11255-022-03103-w)

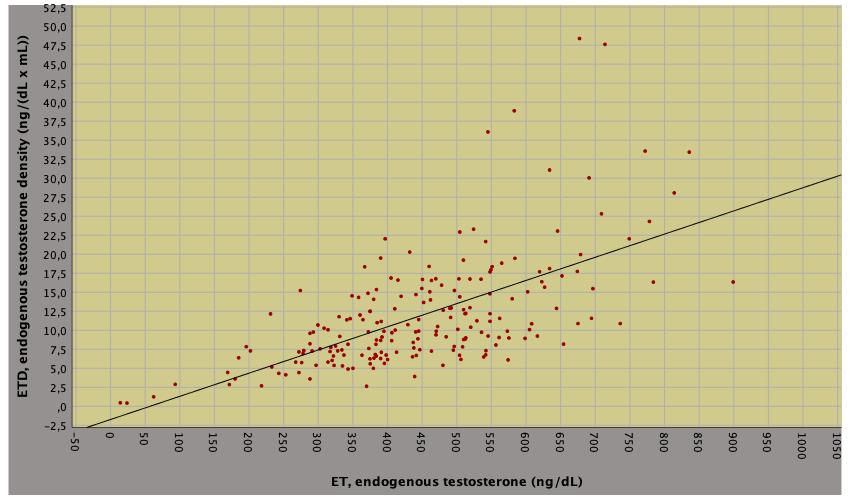

Supplement: Supplementary file 1 — Supplementary file1 Figure S1. Positive correlation between endogenous testosterone (ETD) and endogenous testosterone (ET): as ET levels incremented, ETD values also increased accordingly. ETD was evaluated as the ratio of ET on prostate volume. Pearson’s correlation coefficient, r = 0.619 (p < 0.0001). (JPG 53 KB) [file 11255_2022_3103_MOESM1_ESM.jpg]

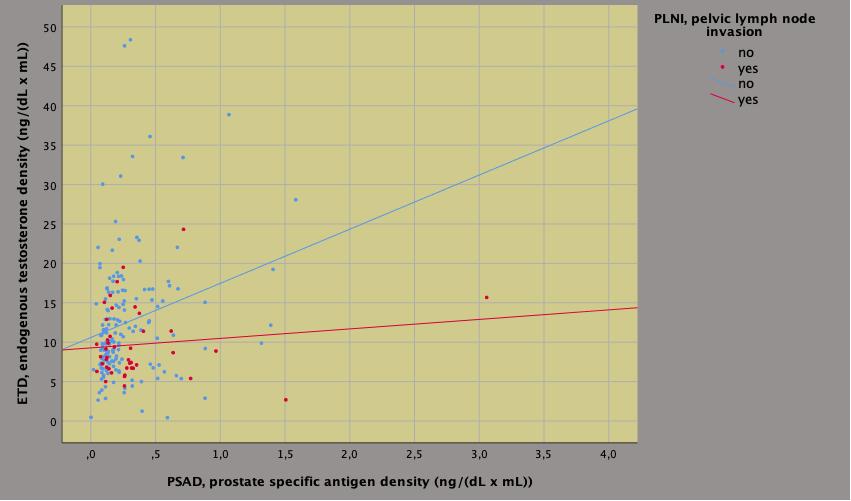

Supplement: Supplementary file 2 — Supplementary file2 Figure S2. Significant positive association of endogenous density (ETD) with prostate specific antigen density (PSAD): as PSAD increased, ETD incremented accordingly but lower ETD values were detected in tumors associating with pelvic lymph node invasion (PLNI), as well. ETD was evaluated as the ratio of endogenous testosterone (ET) on prostate volume (PV). (JPG 36 KB) [file 11255_2022_3103_MOESM2_ESM.jpg]

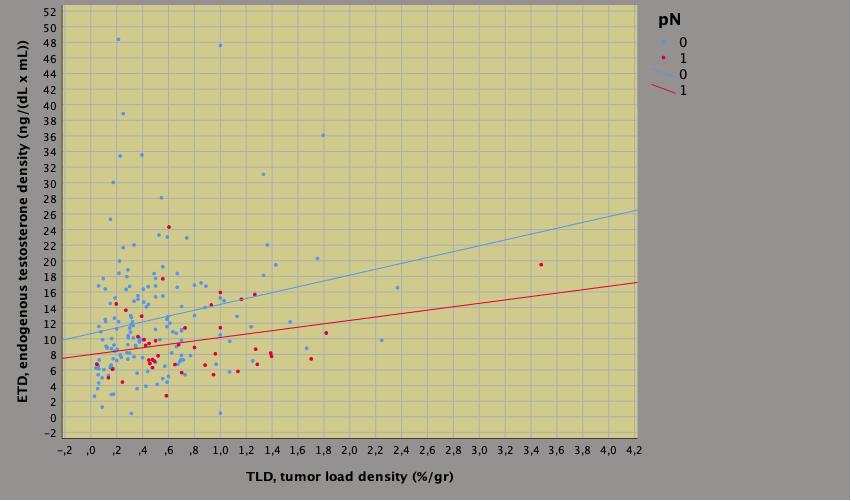

Supplement: Supplementary file 3 — Supplementary file3 Figure S3. Significant positive association of endogenous testosterone density (ETD) with tumor load density (TLD): as TLD incremented, ETD increased accordingly; however, tumors associating with pelvic lymph node invasion (PLNI) showed lower mean levels of ETD when compared with cancers without, as well. ETD was evaluated as the ratio of endogenous testosterone (ET) on prostate volume, while TLD as the ratio of percentage cancer involving the prostate on prostate weight. (JPG 47 KB) [file 11255_2022_3103_MOESM3_ESM.jpg]

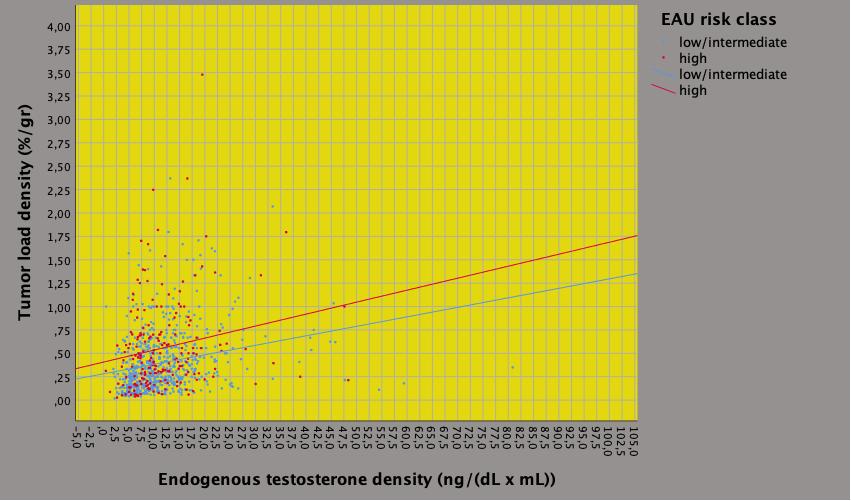

Supplement: Supplementary file 4 — Supplementary file4 Figure S4. The boxplot of the bivariate model of ETD predicting TLD stratified by EAU risk classes (high risk versus low-intermediate classes). As ETD increased, TLD incremented, accordingly, but TLD increments were higher for EAU high risk class; moreover, as shown by the model, for same values of TLD (example TLD = 1), measurements of ETD were lower for the high-risk class, which mean lower mean levels of ET addressing features of aggressive disease. (JPG 60 KB) [file 11255_2022_3103_MOESM4_ESM.jpg]
